# Chlamydial Lipoproteins Stimulate Toll-Like Receptors 1/2 Mediated Inflammatory Responses through MyD88-Dependent Pathway

**DOI:** 10.3389/fmicb.2017.00078

**Published:** 2017-01-26

**Authors:** Yong Wang, Qiong Liu, Ding Chen, Jie Guan, Linghui Ma, Guangming Zhong, Hengping Shu, Xiang Wu

**Affiliations:** ^1^School of Basic Medical Sciences, Xiangya School of Medicine, Central South UniversityChangsha, China; ^2^Department of Cardiovascular Diseases, Xiangya Hospital, Central South UniversityChangsha, China; ^3^Department of Microbiology and Immunology, University of Texas Health Science Center at San AntonioSan Antonio, TX, USA

**Keywords:** Chlamydial trachoma, lipoproteins, immune response, Toll-Like Receptor, cytokine

## Abstract

*Chlamydiae* are very important pathogens which could cause several types of diseases in human, but little is known about its pathogenic mechanism. In order to elucidate host inflammatory response and the signal pathway induced by Chlamydial lipoproteins, the predicted lipoproteins of *Chlamydia trachomatis* were tested for their ability to induce the release of proinflammatory cytokines by mouse macrophages or human TLR (Toll-Like Receptor) expressing cell lines. The results showed that recombinant proteins of *C. trachomatis* D381, D541, D067, and D775 displayed a strong ability to induce the release of IL-8 in TLR expressing cell line. The signal pathways involved TLR1/2 and TLR2/CD14 but not TLR4. Moreover, except D067, the proinflammatory cytokine induction by D381, D541, and D775 required the thioacylation site (cysteine) for lipid modification and the induction was through MyD88-mediated pathway. Our data supported that lipoproteins played a vital role in pathogenesis of *C. trachomatis*-induced inflammatory responses via TLR pathway. It was the first study to characterize other chlamydial lipoproteins after identifying the role of MIP (D541) on pathogenesis of Chlamydial diseases.

## Introduction

*Chlamydiae* are obligate intracellular bacteria which can infect both human and animals, causing a wide range of diseases (Hahn et al., 1991; Centers for Disease Control and Prevention, 1997; Mertz et al., 1998; Kalayoglu et al., 2002; Corsaro and Greub, 2006). Among the species of *Chlamydiae, Chlamydia trachomatis* infects human ocular (serovars A–C) and urogenital/colorectal (serovars D–K and L1–L3) epithelial tissues, causing trachoma (Wright et al., 2008), and sexually transmitted diseases (Sherman et al., 1990; Peterman et al., 2006), respectively. Although, it is known that chlamydia can induce serious damage and proinflammatory immune response after they invade into human epithelia cells (Patton and Kuo, 1989; Kiviat et al., 1990), few details were known about which chlamydial components led to inflammatory diseases. It is unclear about the relationship between the inflammation caused by *C. trachomatis* and the innate immunity of the host yet.

As major components of the bacterial cell wall, lipoproteins played an important role in pathogenesis of many bacterial diseases. However, there was no enough evidence supporting the presence of chlamydial lipoproteins participated in the pathogenic and host response process. One of the lipoproteins characterized in *C. trachomatis* is macrophage infectivity potentiator (MIP), similar to the murein lipoprotein (LppA) from *Escherichia coli* (Bas et al., 2010). Dr. Syvette Bas's group further verified that MIP lipoprotein induced inflammatory response in the pathogenesis of *C. trachomatis* through TLR2/TLR1/TLR6 and CD14 (Bas et al., 2008). Besides MIP and LPS, chlamydial Hsp60 (cHsp60) was another reported molecule responsible for innate immunity via TLR (Toll-Like Receptor) pathway, and cHsp60 acts as the intrinsic ligands of TLRs that evoked the essential signaling pathway of immune responses. Hsp60 activated mononuclear macrophage through TLR4 signal pathway (Ohashi et al., 2000), and Hsp60-mediated B cell initial activation was through TLR4-MyD88 signaling pathway (Flohé et al., 2003; Cohen-Sfady et al., 2005). Bulut found that cHsp60 induced NF-κB activation via TLR4-MD2 and MyD88 pathway as endotoxin and the effect of cHsp60 could be blocked by anti-TLR4 antibody rather than anti-TLR2 antibody (Bulut et al., 2002). Thus, in this study, Hsp60 and LPS were chosen as controls for identifying the unknown chlamydial components involved in TLR4 pathway.

The aim of our study was to illuminate the role of chlamydial lipoproteins in host inflammatory responses of chlamydial diseases. To achieve these aims, we tested the ability of the predicted chlamydial lipoproteins to induce the production of proinflammatory cytokines. As TLRs and CD14 are commonly involved in bacterial lipoprotein recognition (Janeway and Medzhitov, 2002), our experiments were conducted in the cells expressing TLR1/2, TLR2, TLR2/6, and TLR2/CD14 but not TLR4. These receptors are expressed on cell surface of HEK-293 cells which are stably transfected with the empty plasmid (293-Null), human TLR1/2/TLR2/TLR2/6, or TLR2/CD14 genes.

## Materials and methods

### Lipoprotein prediction

The lipoproteins of *C. trachomatis* were predicted using DOLOP (A database of bacterial lipo-proteins). The result can be find at http://www.mrc-lmb.cam.ac.uk/genomes/dolop/predicted/ct.shtml.

### Bacteria and proteins

All chlamydial organisms were either purchased from ATCC (Manassas, VA) or acquired from Dr. Harlan Caldwell at the Rocky Mountain Laboratory, NIAID/NIH and Dr. Ted Kou at the University of Washington (Seattle, WA). The chlamydial organisms were propagated, purified, aliquoted, and stored as previously described (Zhong et al., 2001). All chlamydial organisms were routinely checked for mycoplasma contamination.

### Chlamydial gene cloning and fusion protein expression

The ORFs of D381, D541, D067, and D775 from *C. trachomatis* serovar D were cloned into pGEX-6P-2 vectors (Amersham Pharmacia Biotech, Inc., Piscataway, NJ). According the prediction site of the lipobox, the target amino acid mutagenesis primers were designed and constructed into pGEX-6P-2 vector according the protocol of quick change ^R^ II site directed mutagenesis Kit. The following primers were used for cloning the ORFs:

**Table:** 

CT567 -P1#	5′-CGCGGATCC-ATGTTTAGAAAGATAAAAAAGAA-3′
CT 567-P2#	5′-CCGGAGCTC-TCTACCCACAGCAAAAATATAG-3′
CT 067-P1#	5′-CGCGGATCC-ATGTCTTTTTTTCATACTAGAA-3′
CT 067-P2#	5′-CCGGAGCTC-TTCAAGAACAGTCCCTCCCAAT-3′
CT 381-P1#	5′-CGCGGATCC-ATGTGCATAAAAAGAAAAAAAACATGGAT-3′
CT 381-P2#	5′-CCGGAGCTC-GTTGTTCAAACCCCACTTCTC-3′
CT 775-P1#	5′-CGCGGATCC-ATGAAGATAGGGTTTTGGCGTA-3′
CT 775-P2#	5′-CCGGAGCTC-GGGGACCTCTAAAGGAG-3′
CT 567-P1#(C20A-site mutation)	5′-CTTTCAGAACTTCTCA TCGCTGCCGTGCTTATC AGTTTGCTGCTC-3′
CT 567-P2#(C20A-site mutation)	5′-GAGCAGCAAACTGAT AAGCACGGCAGCGATGA GAAGTTCTGAAAG-3′
CT 067-P1#(C22A-site mutation)	5′-GGACTCTTGTGTTTAG CAGGCGCTTTCTTAATG AACAGCTGTTCC-3′
CT 067-P2#(C22A-site mutation)	5′-GGAACAGCTGTTCATT AAGAAAGCGCCTGCTAA ACACAAGAGTCC-3′
CT 381-P1#(C24A-site mutation)	5′-TGTAGTTTTTGTTTGA CGGGTGCTTTAAAAGAA GGGGGAGACTCC-3′
CT 381-P2#(C24A-site mutation)	5′-GGAGTCTCCCCCTTCT TTTAAAGCACCCGTCAA ACAAAAACTACA-3′
CT 775-P1#(C20A-site mutation)	5′-TGGCGTAGACTGTATG AGGTGTGTTATACTTCTC TTATAGGGGCC-3′
CT 775-P2#(C20A-site mutation)	5′-GGCCCCTATAAGAGA AGTATAACACACCTCAT ACAGTCTACGCCA-3′
CT 775-P1#(C13A-site mutation)	5′-TGGCGTAGACTGTATG AGGTGGCTTATACTTCT CTTATAGGGTGC-3′
CT 775-P2#(C13A-site mutation)	5′-GCACCCTATAAGAGA AGTATAAGCCACCTCAT ACAGTCTACGCCA-3′
CT 775-P1#(C13A20A-site mutation)	5′-TGGCGTAGACTGT ATGAGGTGGCTTATA CTTCTCTTATAGGGGCC-3′
CT 775-P2#(C13A20A-site mutation)	5′-GGCCCCTATAAGA GAAGTATAAGCCACC TCATACAGTCCGCCA-3′

The ORF was expressed as a fusion protein with glutathione- S- transferase (GST) fused to the N-terminuses as previously described (Wu et al., 2011). Expression of the fusion protein was induced with isopropyl-beta-D-thiogalactoside (IPTG; Invitrogen, Waltham, MA) and the fusion proteins were extracted by lysing the bacteria via sonication in a Triton-X100 lysis buffer (1% TritonX-100, 1 mM PMSF, 75 units/ml Aprotinin, 20 μM Leupeptin, and 1.6 μM Pepstatin; Sigma-Aldrich, St. Louis, MO). After removing cell debris by high-speed centrifugation, the fusion protein was purified using glutathione-conjugated agarose beads (Pfizer Inc., New York, NY).

### Cell culture

TLR4^−/−^, myD88^−/−^, Tirap/Mal,^−/−^ Trif^−/−^, and female Balb/c mice at the age of 3–4 weeks old were purchased from Charles River Laboratories, Inc. (Wilmington, MA). The isolated monocytes were seeded in 96-well plates (100 cells/μl) in DMEM supplemented with GlutaMAX-I (2 mM), streptomycin (100 μg/ml), penicillin (100 U/ml), and heat inactivated 10% (v/v) FBS (Invitrogen, Waltham, MA). After the cells were stimulated for 24 h at 37°C, the supernatants were collected by centrifugation (400 × *g*, 4°C, 10 min). The LPS (*E. coli* serotype O55:B5) supplemented in cell culture and triacylated control lipopeptide, racemic Pam3, were purchased from Sigma-Aldrich (St. Louis, MO). Affinity chromatography on polymyxin B agarose (Sigma-Aldrich, St. Louis, MO) was used to remove LPS from recombinant proteins.

### Cell stimulation experiment

Transfected HEK-293 cell lines stably expressing human TLR1/2, TLR2, TLR2/6, or TLR2/CD14 genes were gifts from Dr. Yan Xiang (University of Texas, Health Science Center at San Antonio). The cells carrying an empty plasmid, TLR1/2 plasmids, or TLR2/6 plasmids were cultured in high glucose DMEM (Dulbecco's Modified Eagle's Medium; Invitrogen, Gaithersburg, MD) with 10% FBS, streptomycin (100 μg/ml), penicillin (100 U/ml), and blasticidin S (10 mg/ml; InvivoGen, San Diego, CA). The cells carrying TLR2/CD14 plasmids were cultured in in high glucose DMEM supplemented with 50 mg/ml Hygromycin B Gold (InvivoGen, San Diego, CA). After 24 h culture in a 96-well microplate with an initial concentration of 500 cells/μl, the cells were co-cultured with *E. coli* LPS (1 μg/ml) or lipoproteins (1 to 0.001 μg/ml) for another 24 h. The lipoproteins were diluted in D10 medium (with 20 μg/ml gentamycin and with or without polymycin B 50 μg/ml) and incubated at 37°C for 1 h. The culture supernatants were collected for the ELISA analysis of IL-8 or mMIP-2 levels (DuoSet ELISA; R&D Systems, Inc. Minneapolis, MN). Results were obtained from cultures performed in triplicates.

### Proteinase and lipase treatments of lipoproteins

Proteinase K sensitivity was tested by incubating D775 or D541 with 100 μg/ml proteinase K (Promega) in 100 mM Tris-HCl (pH 8.0) at 37°C for 2 h, followed by addition of 200 mM PMSF (phenylmethylsulfonyl fluoride). Lipoproteins D775 and D541 in a buffer with 50 mM HEPES (pH 7.5) and 10 mM CaCl_2_ were incubated with1000 U/ml pig pancreas lipase (type VI-S lipase; Sigma-Aldrich) at 37°C for 16 h, followed by heating at 100°C before beginning the assay (Bas et al., 2008). The lipoproteins were also incubated in enzyme free buffer as controls. TLR4^−/−^ macrophage were incubated with lipoprotein with lipase, lipoprotein with protease, lipase alone, or protease alone. Cell-free supernatants were harvested after 4 h incubation for cytokine measurement.

### Lipoprotein structure analysis

The lipoprotein structures (D067, D381, D541, and D775) were initially built by homologous modeling using SWISS-MODEL (swissmodel.expasy.org). The initial models were then solvated in a water box and run 10,000 steps of energy minimization and 10 ns molecular dynamics using NAMD (NAnoscale Molecular Dynamics) v 2.9. The modeled lipoprotein-TLR complex structures were built by manually aligning the lipobox cysteine to the Pam3-cysteine based on the crystal structure of TLR2/TLR6- Pam2CSK4 complex (PDB code: 3A79).

## Results

### Fourteen lipoproteins of *Chlamydia trachomatis* were predicted

Fourteen chlamydial lipoproteins were predicted from its genome. They are D067, D175, D253, D381, D386, D444, D541, D548, D567, D600, D734, D770, D775, and D780. The possible function and putative lipobox were also predicted (Table 1). Our previous animal experiments data suggested that CT381 (ABC transporter, solute binding pr; R-binding periplasmic pr 2), CT567 (Hypothetical pr), CT541 (MIP-like pr), CT067 (ABC transporter, solute binding pr; adhesin), CT775 (possible sn-Glycerol-3-P Acyltransferase) were related to the inflammatory reaction. The modeled structures of D067, D381, D541, and D775 were displayed in Supplementary Figure 1. Five proteins were chosen for expression and mutation to explore their roles in host innate immunity.

**Table 1:** **Fourteen predicted ***Chlamydia trachomatis*** (serovar D) lipoproteins using DOLOP program**.

**ORF**	**Possible function**	Putative lipobox^*^
D067	ABC transporter, solute binding protein; adhesin	-LAGC_22_-
D175	Oligopeptide binding protein	-LTSC_23_-
D253	Hypothetical protein	-LSSC_23_-
D381	ABC transporter, solute binding protein; R-binding periplasmic protein 2	-LTGC_24_-
D386	Hypothetic protein (YEY6-like)	-VTAC_24_-
D444	9 kDa Crp	-LSSC_19_-
D541	FkbP-type peptidyl-prolyl cis-trans isomerase (MIP-like protein)	-IVGC_20_-
D548	Hypothetical protein	-LSAC_20_-
D567	Hypothetical protein	-LIAC_20_-
D600	Peptidoglycan lipoprotein	-LSSC_21_-
D734	Hypothetical protein	-LSSC_25_-
D770	β-ketoacyl-ACP synthase	-IVSC_16_-
D775	Possible sn-Glycerol-3-P Acytransferase	-LIGC_20_-
D780	Protein disulfide isomerase	-LIAC_13_-

* *Signal peptidase II cleavage occurs between the lipidated cysteine and its preceding (N-terminal) residue in the lipobox*.

### TLR1/2 and CD14 rather than TLR4 were involved in stimulation of inflammatory cytokine production by *C. trachomatis* lipoproteins

**Figure 1:**
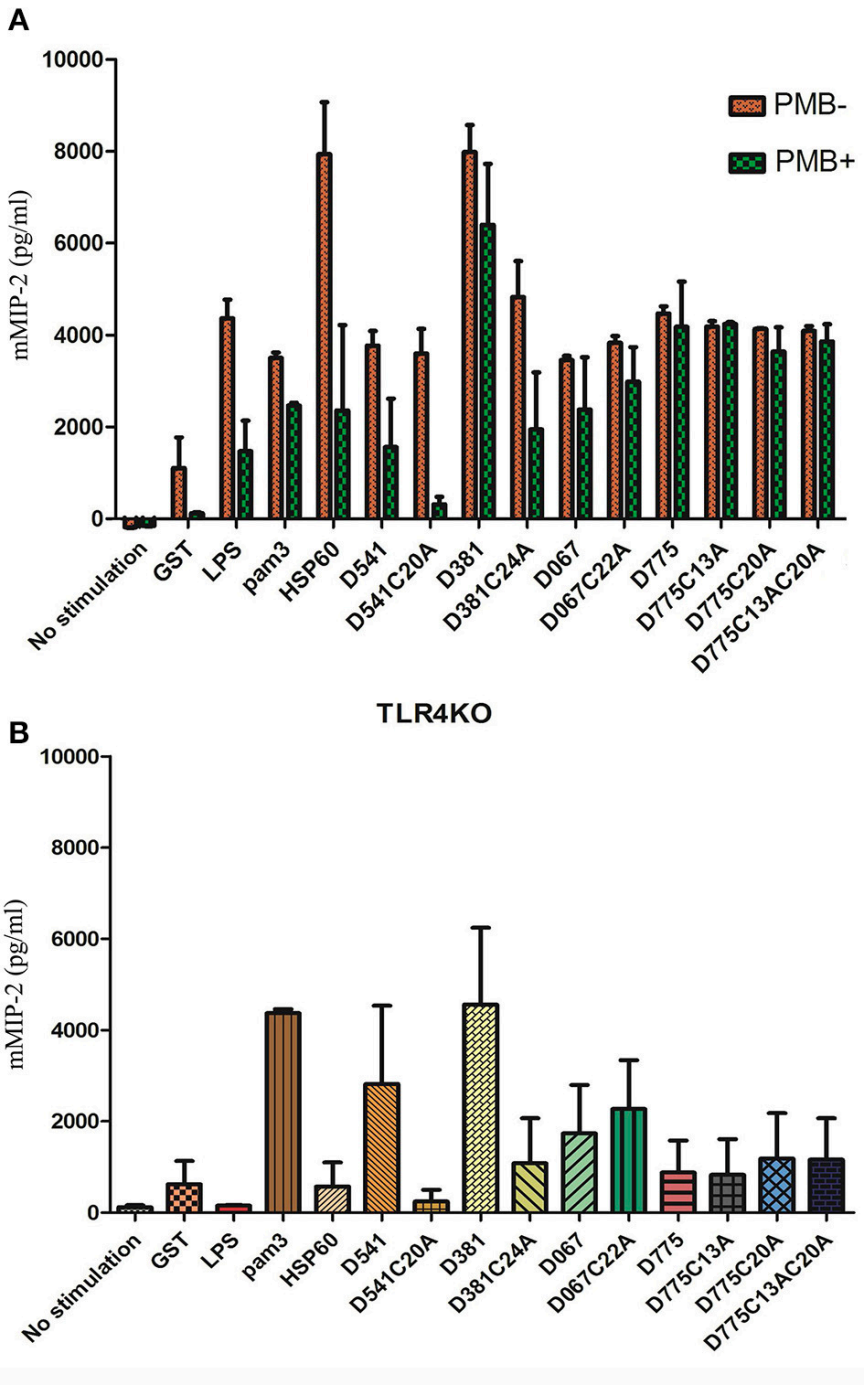
**Cytokine profile of mouse macrophage after ***Chlamydia trachomatis*** lipoprotein stimulation. (A)** Cytokine productions by mouse macrophages in response to lipoproteins of *Chlamydia trachomatis*. Mouse macrophages (10^5^ cells/ml per well) were stimulated by recombinant *C. trachomatis* lipoproteins (D381, D541, D067, D775), lipobox mutated proteins (D381C24A, D541C20A, D067C22A, D775C13A, D775C20A, and D775C13AC20A), Hsp60, *E. coli* LPS, pam3, and GST. The stimulators were incubated with or without polymycin B (50 μg/ml), and supernatants were collected and the cytokine levels were analyzed by sandwich ELISA. **(B)** Cytokine productions by TLR4^−/−^ mouse macrophages in response to lipoproteins of *Chlamydia trachomatis*. Macrophages (10^5^ cells/ml per well) were stimulated by Hsp60, D541, D541 mutant (D541C20A), D381, D381 mutant (D381C24A), D067, D067 mutant (D067C22A), D775, D775 mutants (D775C13A, D775C20A, and D775C13AC20A), *E. coli* LPS, Pam3, and GST. After 24 h culture, supernatants were collected to analyze mMIP-2 levels by ELISA. D381, D541, D067, and D775 could elicit TLR4^−/−^ mouse macrophages to release proinflammatory cytokine, mouse Macrophage Inflammatory Protein-2 (mMIP-2), which was not through TLR4 signal pathway. Except D067, the other 3 proteins required lipobox cysteine for inflammatory cytokine stimulation.

The mouse macrophage were stimulated using *C. trachomatis* recombinant lipoproteins, including D541, D541 mutant (D541C20A), D381, D381 mutant (D381C24A), D067, D067 mutant (D067C22A), D775, D775 mutants (D775C13A, D775C20A, and D775C13AC20A) as well as Hsp60, *E. coli* LPS, Pam3, and GST. Compared to GST and blank control groups, very high levels of mMIP-2 secretion were detected in treatment groups, especially in Hsp60 and D381 treatment groups (Figure 1A). The stimulation effect of LPS decreased by the addition of polymyxin B (PMB), as shown in Figure 1A. The results demonstrated that 50 μg/ml polymycin B could prevent *E. coli* LPS contamination. These data also indicated that the predicted lipoproteins induced proinflammatory cytokines in mouse macrophages.

When the assay were repeated using TLR4^−/−^ mouse macrophage, very few mMIP-2 was detected in LPS and Hsp60 treatment groups while high levels of mMIP-2 were detected in Pam3, D541, D381, D067, and D775 groups (Figure 1B). These result was consistent with previously reported data. However, in the groups of lipobox mutated proteins (D381C24A, D541C20A, D775C13A, D775C20A, and D775C13AC20A), proinflammatory cytokine levels were much lower than the wild type protein groups. It suggested that the stimulation by D541, D381, and D775 required the lipid modification on lipobox cysteine, which was consistent with the lipoprotein prediction results. Therefore, these data indicated that D541, D381, D067, D775 could induce proinflammatory cytokines in macrophages, which might not be only through TLR4 pathway. The stimulation by D541 and D381 required lipid modification. In the presence of lipoproteins, the secretion level of proinflammatory cytokines was correlated with the stimulator concentrations (Figure 1B).

**Figure 2:**
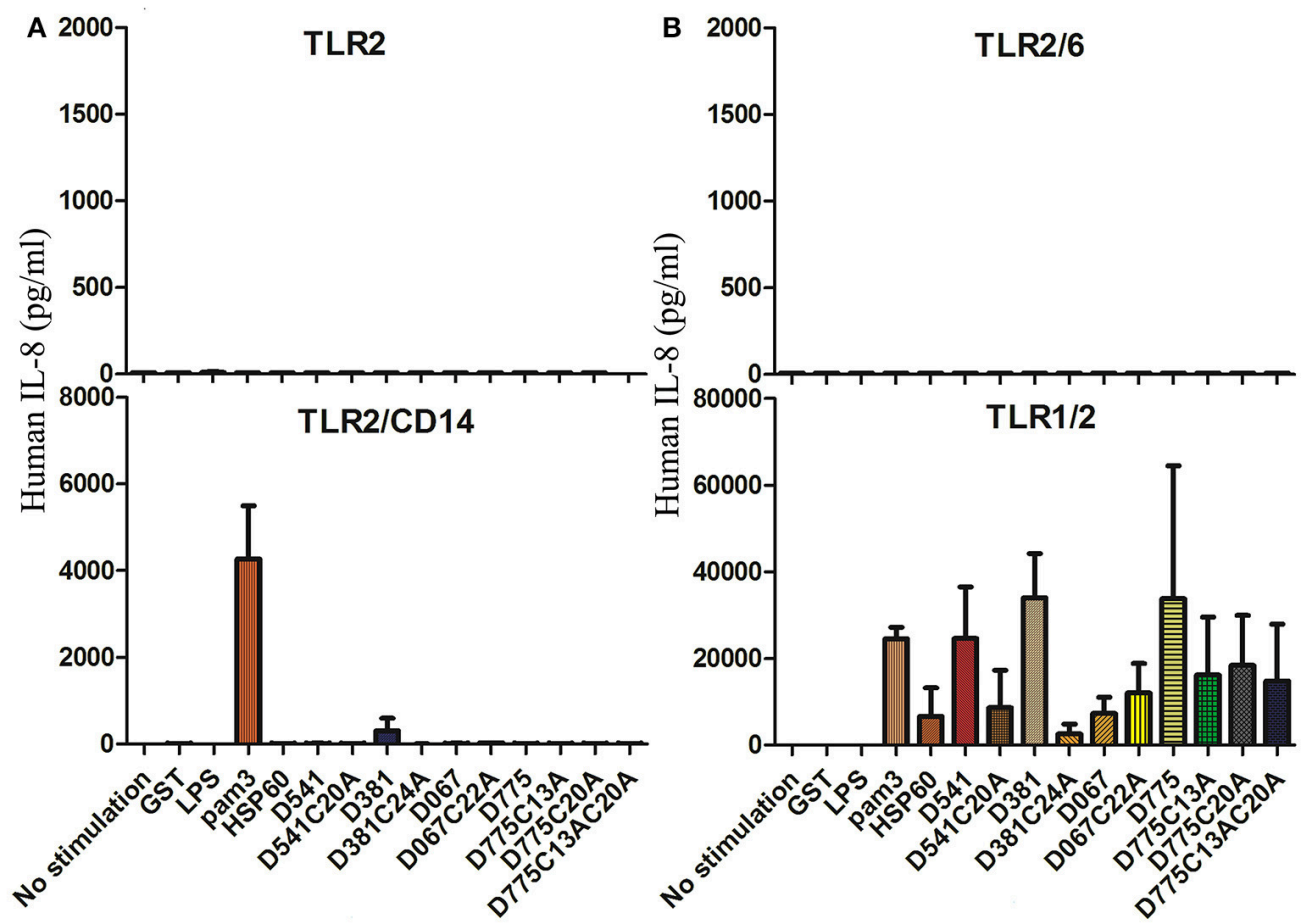
**Toll-Like receptors required for the recognition of chlamydial inflammatory lipoproteins. (A)** IL-8 productions of stimulated TLR2 and TLR2/CD14 expressing cells. **(B)** IL-8 productions of stimulated TLR2/6 and TLR1/2 expressing cells. Cytokine productions of HEK-293 cell lines transfected with human TLR1/2, TLR2, TLR2/6, or TLR2/CD14 genes (10^5^ cells/ml per well) were tested, respectively, after stimulation of *Chlamydia trachomatis* lipoproteins, D541, D541 mutant, D381, D381 mutant, D067, D067 mutant, D775, D775 mutants, Hsp60, *E. coli* LPS, pam3, and GST. The stimulators were diluted in D10 medium with 20 μg/ml gentamycin. After 24 h of culture, supernatants were collected and human IL-8 were analyzed by ELISA.

Cytokine productions were measured for the HEK-293 cells expressing human TLR1/2, TLR2, TLR2/6, or TLR2/CD14 in response to *C. trachomatis* D541, D541 mutant, D381, D381 mutant, D067, D067 mutant, D775, and D775 mutants, as well as Hsp60, *E. coli* LPS, Pam3, and GST. As showed in Figure 2B, high levels of IL-8 were stimulated in TLR1/2 expressing cell line. However, much lower IL-8 levels were detected in their lipobox cysteine mutated proteins compared with that stimulated by wild-type proteins (Figure 2B), suggesting that TLR1/2 signal pathway was involved in Pam3, D541, D381, and D775 stimulations and the stimulation required the lipobox cysteine. There were very low cytokine levels detected in stimulation assays of TLR2 and TLR2/6 expressing cells (Figures 2A,B). In addition, TLR2/CD14 could be stimulated by D381 and Pam3. The results indicated that most of the *C. trachomatis* lipoproteins stimulated proinflammatory cytokines through the TLR1/2 pathway and D381 could also use TLR2/CD14 pathway.

### The induction of proinflammatory cytokines by D775 required lipid modification

**Figure 3:**
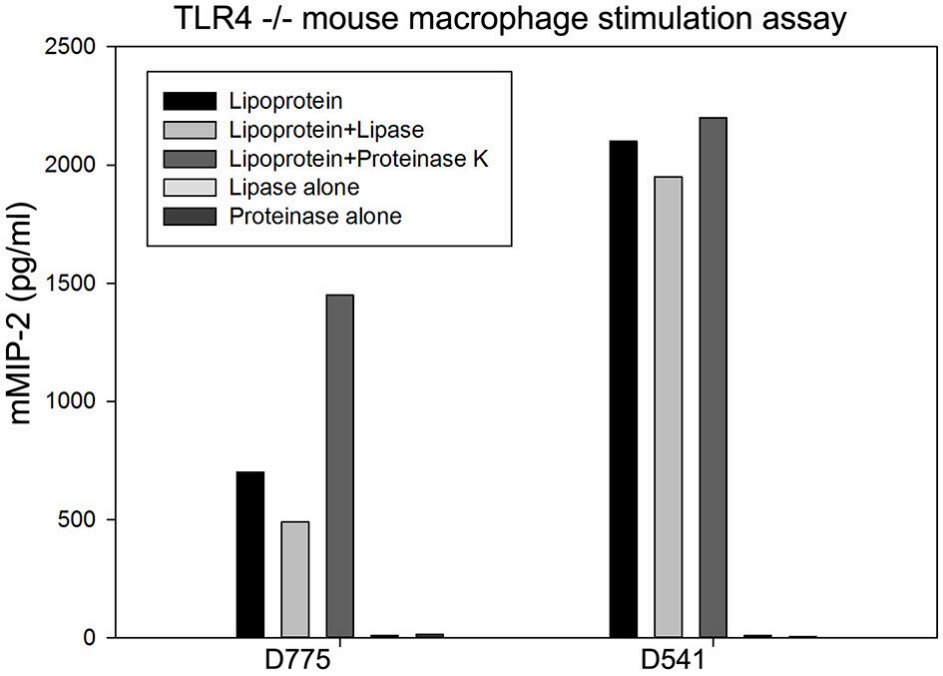
**Production of proinflammatory cytokines induced by D775 required lipid modification while D541 did not**. TLR4^−/−^ mouse macrophage were incubated with lipoproteins treated with lipase, lipoproteins treated with proteinase, lipase alone, or proteinase alone. Lipoprotein+lipase groups: D775 and D541 were incubated with1000 U/ml pig pancreas lipase at 37°C for 16 h, followed by heating at 100°C before beginning cytokine stimulation assay. Lipoprotein+ proteinase groups: D775 or D541 with 100 μg/ml proteinase K at 37°C for 2 h, followed by addition of 200 mM PMSF. Lipoprotein groups: D775 and D541 were incubated in enzyme free buffer as controls. Cell-free supernatants were harvested after 4 h incubation for cytokine mMIP-2 measurement by ELISA. It confirmed that lipid modification of D775 was essential for its stimulation of proinflammatory cytokine production, but D541 stimulation was not greatly affected by lipase.

To explore whether the lipid or protein part contributes to the proinflammatory activity of recombinant lipoproteins, D775 and D541 were first treated with proteinase K. Results showed that proinflammatory activity of D775 and D541 was maintained after proteinase digestion (Figure 3), which was similar to the result of a macrophage-activating lipopeptide from *Mycoplasma fermentans* (Mühlradt et al., 1996). However, lipase treatment of Chlamydial lipoprotein D775 led to dramatic decrease of mMIP-2 release after cell stimulation. These results confirmed that D775 required lipid modification to stimulate proinflammatory cytokine production. But our data indicated that D541 did not significantly influenced by lipase which was inconsistent with a previous report (Bas et al., 2008).

### Production of proinflammatory cytokines induced by D775 and D541 was through MyD88-dependent pathway

**Figure 4:**
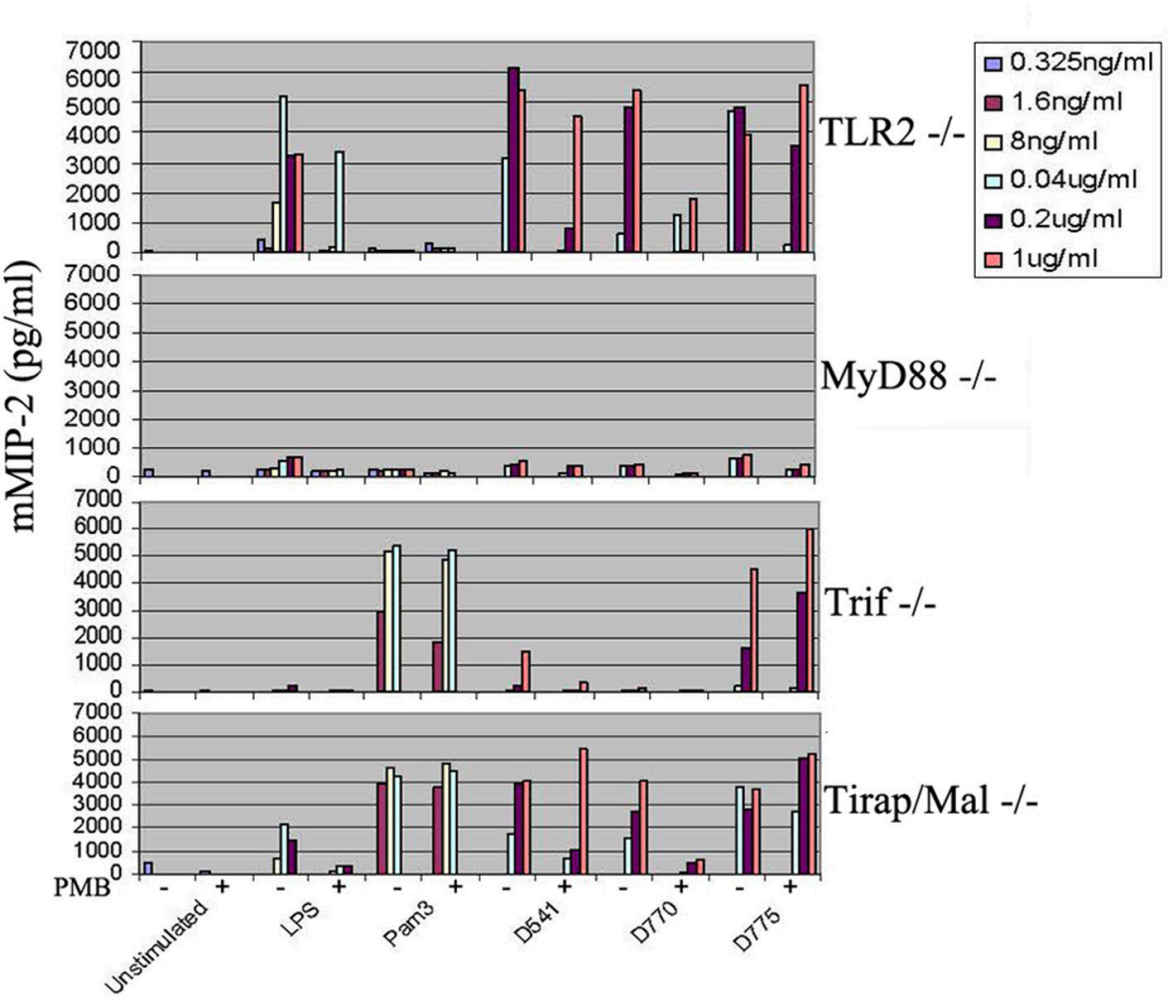
**Cytokine productions by myD88^**−/−**^, Tirap/Mal^**−/−**^, and Trif^**−/−**^mouse macrophages in response to ***Chlamydia trachomatis*** lipoproteins**. The mouse macrophages (10^5^ cells/ml per well) were stimulated by recombinant *C. trachomatis* lipoproteins D541 and D775 as well as *E. coli* LPS and Pam3, and then mMIP-2 level was determined by ELISA. The “+” symbol represents the addition of polymycin B (50 μg/ml) while the “−” symbol in the figure indicates that no polymycin B is supplemented. After 24 h culture, supernatants were collected and the mMIP-2 were analyzed. Results were obtained from 6 different dilution concentrations of stimulators and cell cultures performed in triplicates. The results suggested the proinflammatory cytokine production induced by D775 and D541 relied on MyD88 while D541 stimulation depended on both MyD88 and Tirap/Mal.

To determine whether the ability of proinflammatory cytokine production induced by D541 and D775 was in a MyD88-dependent manner, cytokine productions in MyD88 KO, Tirap/Mal KO, and Trif KO macrophages were stimulated by *C. trachomatis* recombinant lipoproteins (D541 and D775), LPS, and Pam3. When MyD88 were knocked out, mMIP-2 production decreased to an extreme low level in the stimulation assay of LPS, Pam3, D541, and D775 (Figure 4). It showed that the proinflammatory cytokines induced by D775 and D541 were through MyD88-dependent pathway. When Tirap/Mal were knocked out, mMIP-2 release remained high level after stimulation by Pam3 and D775 while the stimulation of D541 relied on both MyD88 and Tirap/Mal. The knock-out of Trif substantially decreased the stimulation effects of D541.

## Discussion

Chlamydia infection can cause high levels of inflammatory cytokines in the culture supernatants or at the site of infection. However, it is unclear how chlamydial organisms activate inflammatory cytokine genes and what chlamydial components are responsible for or participate in the host cytokine gene activation. Therefore, looking for chlamydial lipoproteins that activate the host cells cytokine secretion has become an interesting topic in chlamydia research field. To date, the only study of chlamydial lipoprotein was the D541 (MIP) which induces cytokines release in a TLR4-independent fashion (Bas et al., 2008).

TLRs are a class of receptors that play a key role in the innate immune system and initiating the inflammatory reactions (Lien et al., 1999; Akira and Takeda, 2004; Kufer and Sansonetti, 2007). They are single, membrane-spanning, non-catalytic receptors usually expressed in sentinel cells such as macrophages and dendritic cells and recognize structurally conserved molecules derived from microbes. It has been verified that different TLRs recognized PAMPs by matching the steric configuration of the ligands in TLR knock out mouse. TLR4 recognizes LPS while TLR2 recognizes bacterial lipoprotein and glycolipid. TLR2/TLR1 heterodimers bind triacylated lipopeptides, whereas TLR2/TLR6 heterodimers recognize diacylated lipopeptides (Takeuchi et al., 2001; Takeda et al., 2002; Akira, 2003).

Our study demonstrated that among the 14 predicted lipoproteins of *C. trachomatis*, D381, D541, D067, and D775 have displayed greater ability of proinflammatory cytokine induction. Their proinflammatory activity could not be attributed to *E. coli* LPS contamination since the induction was insensitivity to polymyxin B. The induction may involve TLR1/2 and TLR2/CD14, but not TLR4 pathway. These results are consistent with previous reports of the predominant role of TLR2 in C. *trachomatis* inflammatory reaction (Darville et al., 2003). They demonstrated that TLR2 was directly involved in the disease development since there was no distinct pathologic change in oviduct after *C. trachomatis* infection in TLR2-deficient mice (Darville et al., 2003; He et al., 2011). On the other hand, CD14 could enhance TLR2-mediated responses to many PAMPs including peptidoglycans and lipopeptides. Therefore, it is postulated that CD14 participated in transferring signal from these ligands to TLR2 (Sellati et al., 1998; Wetzler, 2003; Akira and Takeda, 2004; Vasselon et al., 2004), which was the first step of the activation and transduction of immune response signals (Tsan and Gao, 2009; Casanova et al., 2011).

In TLR signal system, there are 4 adaptor proteins including Mal (MyD88 adaptor- like protein), TRIF (Toll-interleukin-1 receptor domain-containing adaptor inducing interferon-γ), TRAM (Trif-related adaptor molecule), and SARM (Sterile and HEAT-Armadillo motifs) which participated in TLR-associated signaling cascades. It has been proved that the interaction among TRAM and TRIF, Mal, TLR4 could affect the function of non-MyD88-dependent TLR4 signal pathway (Fitzgerald et al., 2003). In our study, results showed that the proinflammatory cytokines induced by D775 and D541 were through MyD88-dependent pathway. Most of our results on D541 were consistent with the results from Dr. Sylvette Bas's group (Bas et al., 2008). Furthermore, we found that the induction by D541 and Pam3 was partially relied on MyD88 and Tirap/Mal.

Except D067, the stimulatory activities of D381, D541, and D775 required lipid modification. Their effects on cytokine release were greatly decreased when the lipobox cysteines were mutated. In addition, cytokine induction activity of D775 was greatly reduced by lipase treatment, but proteinase K treatments did not affected its activity. It was strange that the stimulation effect of D541 was not significantly decreased by lipase treatment. This was contradicted with Sylvette Bas's results. The detail mechanism required further studies.

To gain the insight of structural characteristics of *C. trachomatis* lipoproteins, the homologous protein structures of D067, D381, D541, and D775 were predicted by homologous sequence searching (Supplementary Figure 1). The homolog of D541 is a peptidyl-prolyl cis-trans isomerase which could serve as the ligand of TLR. For example, a peptidyl prolyl cis-trans isomerase, HP0175, secreted by *Helicobacter pylori* is able to TLR4-dependently induce apoptosis, and could also be specifically pulled down by TLR4 (Basak et al., 2005).

In conclusion, the predicted Chlamydial lipoproteins D381, D541, D067, and D775 are very important factors in host inflammatory responses to the diseases caused by *C. trachomatis* via TLR pathways. This is the first exploring study on the effect of Chlamydial lipoproteins on pathogenesis of Chlamydial diseases after the report of MIP (D541). This study may provided the theoretical basis for drug development, molecular modification of vaccine and the prevention and cure of the diseases caused by *C. trachomatis*.

## Author contributions

YW and QL finished parts of the experiment and did the statistics. DC, JG, and LM contributed to experimental technique supports. GZ and HS provided much scientific advice for this study. XW finished parts of the experiments and wrote the paper.

### Conflict of interest statement

The authors declare that the research was conducted in the absence of any commercial or financial relationships that could be construed as a potential conflict of interest.

## Supplementary material

Supplementary Figure 1 **The modeled protein structures of chlamydial inflammatory lipoproteins**. The predicted 3-dimensional structures of *Chlamydia trachomatis* lipoproteins are listed in different panels. **(A)** The structure model of D541. **(B)** The structure model of D067-Toll like receptor complex. **(C)** The structure model of D381. **(D)** The structure model of D775-Toll like receptor complex.
