# Supplementary figures and images for: Chlamydial Lipoproteins Stimulate Toll-Like Receptors 1/2 Mediated Inflammatory Responses through MyD88-Dependent Pathway
Source: Front Microbiol. 2017 Jan 26;8:78. doi: 10.3389/fmicb.2017.00078 (PMC5266682; doi:10.3389/fmicb.2017.00078)

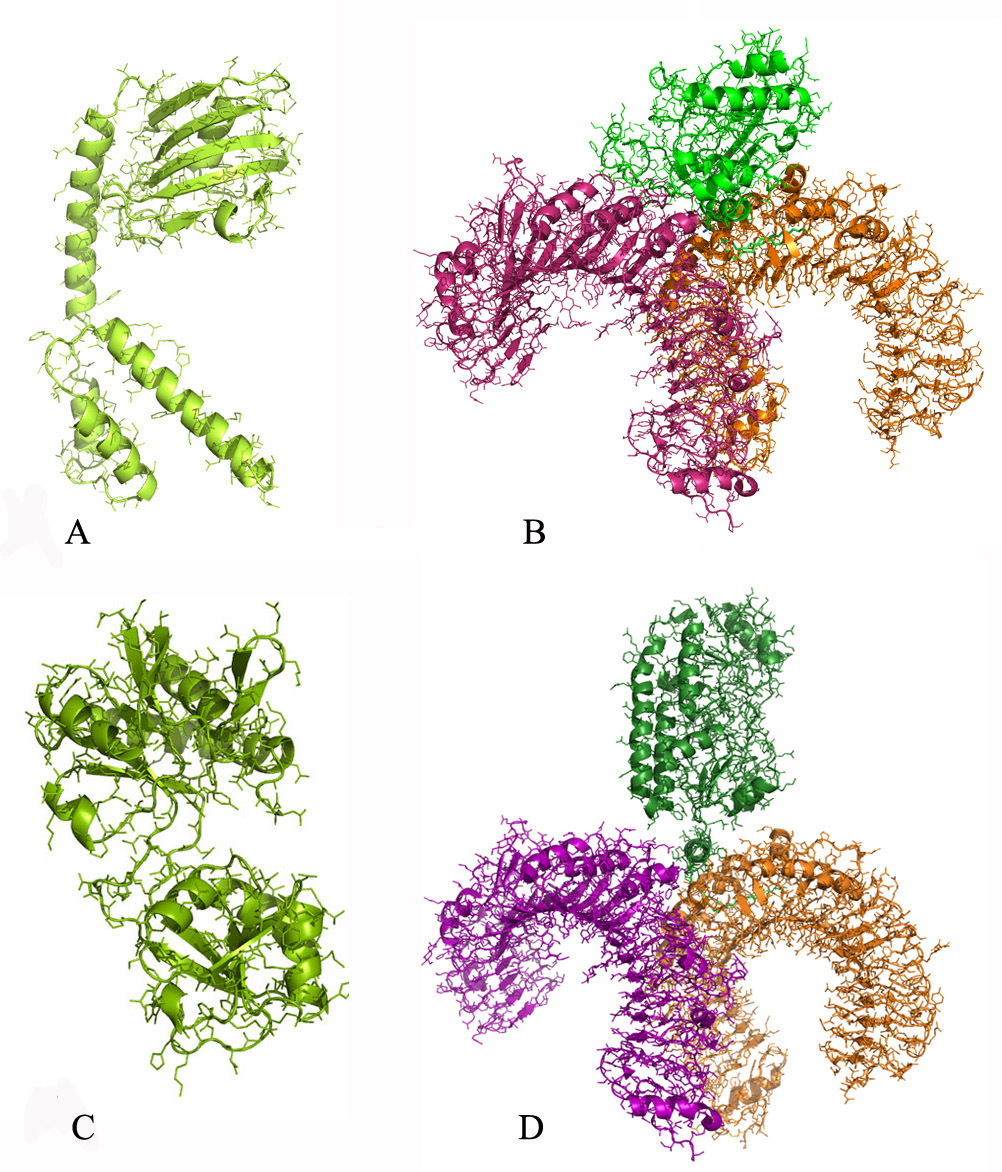

Supplement: Supplementary Figure 1 — The modeled protein structures of chlamydial inflammatory lipoproteins. The predicted 3-dimensional structures of Chlamydia trachomatis lipoproteins are listed in different panels. (A) The structure model of D541. (B) The structure model of D067-Toll like receptor complex. (C) The structure model of D381. (D) The structure model of D775-Toll like receptor complex. [file Image1.JPEG]
